# Solitary Angiokeratoma of the Labial Mucosa: Report of a Rare Case and Literature Review

**DOI:** 10.3390/dj10020017

**Published:** 2022-01-21

**Authors:** Rahaf M. Alhazmi, Najla Dar-Odeh, Hamzah Babkair

**Affiliations:** 1 College of Dentistry, Taibah University, Al-Madinah Al-Munawwarah 42353, Saudi Arabia; najla@ju.edu.jo (N.D.-O.); Hbabkair@taibahu.edu.sa (H.B.); 2 School of Dentistry, University of Jordan, Amman 11942, Jordan

**Keywords:** solitary angiokeratoma, labial mucosa, oral cavity

## Abstract

Oral angiokeratoma is a rare vascular lesion that has various clinical presentations. It usually occurs as part of generalized angiokeratoma and rarely appears as a solitary lesion with no underlying systemic diseases. Only 33 cases were reported so far worldwide. In this case report, we present a rare case of isolated solitary oral angiokeratoma in a 22-year-old female patient, which is the first case to be reported in the labial mucosa that has been treated successfully by surgical excision.

## 1. Introduction

Angiokeratoma (AK) is a rare congenital or acquired mucocutaneous vascular lesion caused by dilated vessels lying in the superficial dermis and accompanied by epidermal reaction [1,2]. The estimated worldwide prevalence is 0.16% [3]. Clinically, it can manifest as a localized or generalized lesion, and it can be solitary or multiple. It can affect patients with underlying systemic disease, as well as healthy individuals. The most common form is solitary angiokeratoma of the lower extremities, which arises after a history of trauma. Other forms include Fordyce-type angiokeratoma, which is limited to scrotum or vulva; Mibelli-type angiokeratoma that appears on bony prominences as a bilateral lesion with a history of trauma; Angiokeratoma circumscriptum, which is the congenital and the least common form of AK; lastly, generalized angiokeratoma, known as angiokeratoma corporis diffusum, which is usually associated with metabolic disorders such as Fabry’s disease and fucosidosis [4,5,6]. It should be noted that all types of AK have the same histological features [7].

Oral angiokeratoma (OAK) is a very rare lesion that usually occurs as part of a generalized angiokeratoma. However, solitary OAK that occurs as a solitary event is considered to be one of the rarest presentations of AK [1,4]. To the best of our knowledge, only 33 cases have been reported in the literature intraorally with the tongue being the most commonly affected site. Therefore, we report this rare case of isolated solitary OAK in a 22-year-old female patient, which is considered to be the first case that has been reported in the labial mucosa. 

## 2. Case Presentation

**Figure 1:**
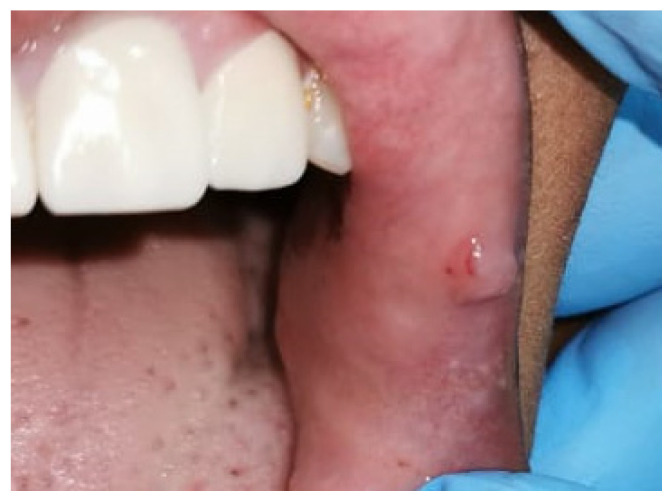
A preoperative photograph showing a single pink papule with petechial hemorrhages on the upper labial mucosa.

A 22-year-old female patient presented to Taibah University Dental Hospital complaining of a swelling on the left inner side of the upper lip with intermittent bleeding and painful episodes. The onset was three years ago when the patient was undergoing a fixed orthodontic treatment. The lesion started as a painful ulcer caused by repeated trauma from a sharp orthodontic appliance. A few months later, a painless papule developed. There were intermittent episodes of inflammation, pain, and bleeding upon exposure to trauma or accidental lip biting. Her medical history was free of any systemic diseases or medication consumption. Upon extraoral examination, right and left submandibular lymph nodes were palpable, mobile, and non-tender. Intraoral examination revealed a 0.8 × 0.6 cm pink, soft, non-tender, well-circumscribed papule, with some petechial hemorrhages, and it is located on the left upper labial mucosa 0.1 cm away from the vermillion border and near to the oral commissure (Figure 1). No other lesions were detected elsewhere. 

**Figure 2:**
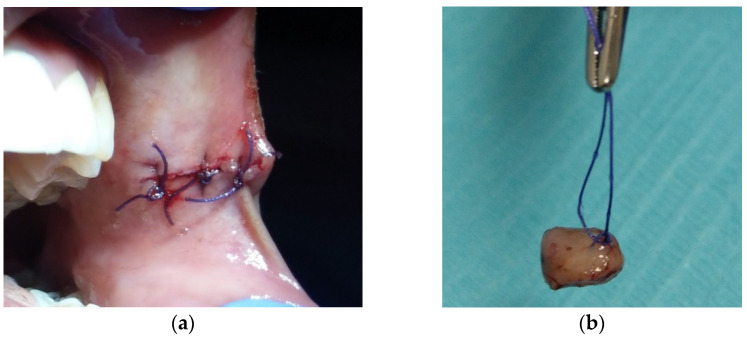
Intraoperative photographs: (**a**) surgical defect was closed using 3/0 absorbable braided polyglactin suture; (**b**) the specimen was oriented with a suture in its superior border.

Based on the history and clinical appearance, the preliminary differential diagnoses were irritation fibroma, neurofibroma, and pleomorphic adenoma. Since there were no signs of malignancy, surgical excision with a safety margin was performed after anesthetizing the area with 2 mL of 2% lidocaine with 1:100,000 epinephrine, to remove the lesion and establish a histopathological diagnosis. The surgical defect was closed using a 3/0 absorbable braided polyglactin suture (Figure 2a). The specimen measured 0.8 × 0.6 × 0.3 cm and was oriented with a suture in its superior border and then was placed in a labeled container of formalin with patient identification (Figure 2b).

**Figure 3:**
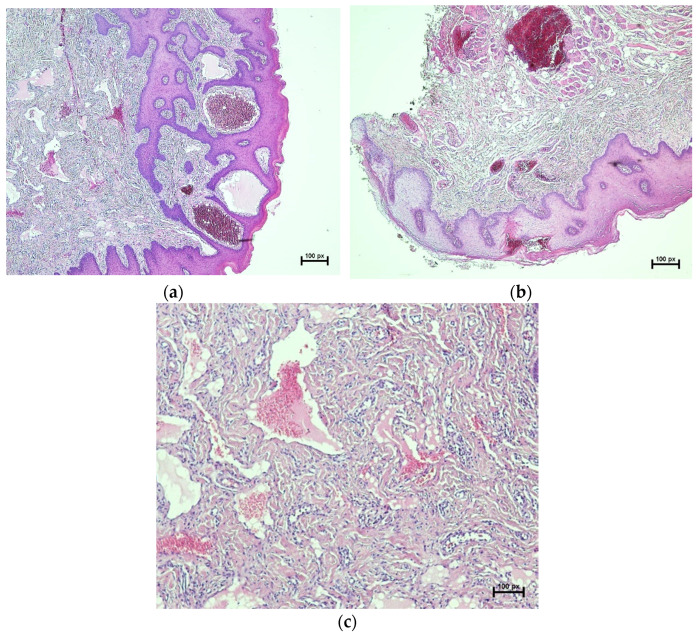
Histopathological findings of AK. HE stains, original magnification X4: (**a**) increase in keratin layer, acanthosis, and papillary-like projection of epithelial layer with multiple dilated vascular vessels surrounded by papillary projection; (**b**) multiple dilated vascular vessels filled with RBCs and surrounded by connective tissues; (**c**) dilated vascular vessels are surrounded by muscle layers and filled with RBCs.

**Figure 4:**
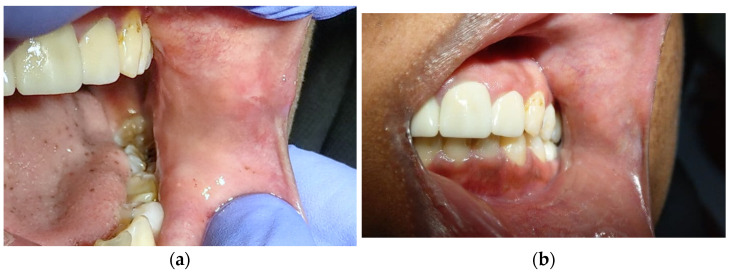
(**a**,**b**) Postoperative photograph of the surgical site after one month, showing normal healing without scarring.

Histological examination revealed an increase in the keratin layer, acanthosis, and papillary-like projection of epithelium. Beneath the epithelial layer, there were multiple dilated vascular vessels filled with red blood cells and surrounded by papillary projection and muscle layer (Figure 3). Based on the histological findings, a diagnosis of AK was established. The final diagnosis was confirmed to be isolated solitary OAK. After one month, the patient came for follow-up and the surgical site showed normal healing without scarring (Figure 4a). Two years later, the patient was reviewed, and there was no evidence of recurrence (Figure 4b).

## 3. Discussion

In 1889, the first case of AK was reported by Mibelli V on fingers and toes [8]. Almost 100 years later in 1997, Leung CS reported the first case of solitary isolated OAK on buccal mucosa that affected an 82-year-old male [9]. OAK is a rare lesion that usually presents as part of Fabry’s disease or fucosidosis. It can be a congenital lesion and, in this case, it is considered as angiokeratoma circumscriptum. Isolated OAK with no underlying systemic involvement is a very rare lesion [4]. To the best of our knowledge, there were only 33 cases of isolated OAK that have been reported in the literature so far (Table 1).

As shown in (Table 1), the tongue is the most common site of isolated OAK, with 29 cases being exclusively affecting the tongue, while three cases have been reported with buccal mucosa involvement and only one in the tonsillar pillar. AK is known for being more predominant in females [1,4]. However, our review reveals that isolated OAK is more predominant in male patients, with 19 cases out of 33 cases that have been reported in male patients. Therefore, our case is considered a unique presentation of isolated OAK, which is the first case that has been reported with labial mucosa involvement, and it affected a female patient. 

Clinically, OAK has various clinical presentations that can be single or multiple, reddish to bluish, and it can range in size from a couple of millimeters to several centimeters. Due to its variant clinical presentation, it can be misdiagnosed as hemangiomas, lymphangiomas, or hematomas, as well as malignant melanomas [4]. As OAK has a low frequency and several presentations, its diagnosis is not straightforward, and usually, multiple differential diagnoses are established. It was suggested that OAK is probably more frequent than reported, but due to its clinical presentation, it is usually misdiagnosed [7,23]. In our case, it had a similar presentation to irritation fibroma, neurofibroma, and pleomorphic adenoma. Therefore, histopathological analysis is needed to establish an accurate and definitive diagnosis [4,14]. However, if the diagnosis was confirmed, it is advisable to perform a full examination of the skin and mucosa, searching for other similar lesions and performing the needed investigation to rule out the presence of underlying systemic diseases [14].

Histologically, OAK shows hyperkeratosis and acanthosis of the epithelium with dilated vascular vessels in the sub-epithelial tissues. Cutaneous and oral AK have similar histological pictures except that hyperparakeratosis is seen in oral lesions, while hyperorthokeratosis is seen in cutaneous lesions [1,11]. It has been suggested that the reactive epidermal or epithelium growth is due to the increased proliferative activity on the surface of vascular malformations that are near to the epidermis or epithelium [12]. In our case, the absence of mass of fibrous connective tissue excluded the irritation fibroma. Additionally, there were no signs of tumor in peripheral nerves, and this excluded the neurofibroma. Moreover, pleomorphic adenoma is reported more frequently in the upper lip than the lower lip [35], which is why it was suspected that this lesion was originally pleomorphic adenoma. However, the histological findings ruled out other differential diagnoses and confirm the diagnosis of solitary OAK [36].

OAK is believed to be due to acute or chronic trauma, high venous blood pressure, or vascular malformation. However, the exact pathogenesis is still unknown [5,13]. We suggest that repeated and chronic trauma by the orthodontic appliance is the cause of OAK appearance in our case. 

In 2009, Ranjan and Mahajan proposed the first classification of OAK (Table 2) [37]. Based on their classification, our case is considered type 1As solitary. 

OAK is usually asymptomatic, and there is no need for any treatment once the diagnosis is confirmed, but sometimes, the lesion causes bleeding, discomfort, or cosmetic changes that necessitate intervention [16,27]. In a case reported by Görkem Eskiizmir (2011), no intervention was carried out for the reported multiple OAK in the tongue, and the lesion was observed for one year with no evidence of any changes in the lesion [3]. Based on our review, the first and most common therapeutic option of OAK is surgical excision, to remove the lesion and confirm the diagnosis simultaneously [4]. Other available treatment options are diathermy [4], radiotherapy [2], intralesional steroids administration [17], and laser ablations [1,17,20,24], which can be used separately or in combination with each other. Several types of lasers were proposed in the management of OAK such as carbon dioxide laser [1,17,20], pulsed dye laser [20], diode laser [24]. It has been suggested that for smaller lesions surgical excision, cryotherapy, or electrocautery are preferred, while for larger lesions, it is preferred to use a wide surgical excision and/or laser ablation. However, this can cause significant scarring, especially with Argon laser and Nd: YAG laser [2]. All these treatment options have been reported to manage OAK successfully without recurrent lesions [4]. However, Farooq (2005) reported a case of a recurrent OAK located in the tongue that was being treated by pulsed dye laser [30]. Then, Green and Roy (2006) reported another case of OAK recurrence after an unclarified type of laser treatment was used [28]. Based on their study, they concluded that laser treatment is less effective in treating AK that affects the oral cavity than that affecting the skin, and they recommended surgical excision as the treatment of choice in the case of OAK [28,30]. In this case, OAK was managed by surgical excision and followed up for 2 years with no signs of recurrence.

This study has limitations. It would have been more informative to supplement histopathological examination with immunohistochemical staining for VEGF, VEGFR, MMP9, CD34, CD31, CD3, and CD8 T cell markers. Due to their unavailability, these diagnostic methods were not conducted. However, better insight could have resulted if these tests were conducted to confirm the diagnosis and exclude other causes of proliferative vascular lesions.

## 4. Conclusions

In this paper, we reported the first case of solitary OAK of the labial mucosa that was not accompanied by any underlying systemic disorders or other cutaneous lesions. The lesion was successfully treated surgically with no recurrence detected. This case report confirms the benign nature of angiokeratoma and highlights the possibility that oral angiokeratoma can appear as an isolated mass, and the most appropriate treatment is surgical excision.

## Figures and Tables

**Table 1:** Review of the literature on isolated oral angiokeratoma.

Year	References	Age	Gender	Site	Management
2021	Bins, A. [10]	41	Female	Tongue	Surgical excision
2020	Vizotto, L. M. [11]	39	Male	Tongue	Surgical excision
2019	Gomaa, M. [4]	9	Male	Tongue	Diathermy
2019	Hamid, R. [1]	16	Female	Tongue	Carbon dioxide laser
2018	Kumar, K. S. [12]	11	Male	Tongue	Surgical excision
2017	Bakshi, S. S [5]	9	Male	Tongue	Surgical excision
2016	Job, A. M. [13]	12	Male	Tongue	Surgical excision
2014	Vijay, M. K. [14]	26	Male	Tongue	Surgical excision
2014	Andreadis, D. [7]	45	Female	Buccal mucosa	Surgical excision
2014	Kang, Y. H. [6]	18	Female	Buccal mucosa	Surgical excision
2013	Shah, S. S. [15]	18	Male	Tongue	Surgical excision
2013	Erkal, E. Y. [2]	67	Female	Tongue	Radiotherapy
2013	Kandalgaonkar, S. [16]	38	Male	Tongue	Surgical excision
2012	Patigaroo, S. A. [17]	16	Female	Tongue	Surgical excision, carbon dioxide laser, and Intralesional steroids
2012	Aggarwal, K. [18]	10	Male	Tongue	Not reported
2012	Nain, M. [19]	11	Female	Tongue	Surgical excision
2011	Eskiizmir, G. [3]	66	Female	Tongue	No intervention with close follow-up
2011	Kar, H. K. [20]	12	Male	Tongue	Carbon dioxide laser and pulsed dye laser
2010	Ravi, G. C. [21]	7	Male	Tongue	Surgical excision
2010	Fernandez-Acenero, M. J. [22]	61	Female	Tongue	Surgical excision
2009	Fernandez-Flores, A. [23]	68	Male	Tonsillar pillar	Surgical excision
2009	Ergun, S. [24]	16	Female	Tongue	Diode laser
2008	Sion Vardy, N. [25]	45	Female	Tongue	Surgical excision
2007	Yildirim, M. [26]	9	Female	Tongue	Not reported
2006	Siponen, M. [27]	54	Female	Tongue	Surgical excision
2006	Green, J. B. [28]	6	Male	Tongue	Surgical excision
2005	Varshney, S. [29]	12	Female	Tongue	Surgical excision
2005	Farooq, U. [30]	6	Male	Tongue	Surgical excision
2003	Vijaikumar, M. [31]	12	Male	Tongue	Not reported
2002	Hoshino, M. [32]	40	Male	Tongue	Surgical excision
2001	Bhargava, P. [33]	5	Male	Tongue	Not reported
1998	Kumar, M. V. [34]	16	Male	Tongue	Not reported
1997	Leung, C. S. [9]	82	Male	Buccal mucosa	Surgical excision

**Table 2:** Oral angiokeratoma classification.

**Type 1: Primary (Purely Mucocutaneous without Systemic Disorders)**					
Type 1A: isolated angiokeratomas of the oral cavity		Type 1B: mucocutaneous angiokeratomas (oral and cutaneous)		Type 1C: angiokeratomas occurring simultaneously in oral cavity, skin, and gastrointestinal mucosa	
Type 1As (solitary)	Type 1Am (multiple)	Type 1Bs (solitary)	Type 1Bm (multiple)	Type 1Cs (solitary)	Type 1Cm (multiple)
**Type 2: Secondary (as a Component of a Generalized Systemic Disorder)**					
Type 2A: a component of Fabry’s disease			Type 2B: a component of fucosidosis		
Type 2As (solitary)	Type 2Am (multiple)		Type 2Bs (solitary)	Type 2Bm (multiple)	

## Data Availability

Not applicable.
